# Visualizing Street Pavement Anomalies through Fog Computing V2I Networks and Machine Learning

**DOI:** 10.3390/s22020456

**Published:** 2022-01-08

**Authors:** Rogelio Bustamante-Bello, Alec García-Barba, Luis A. Arce-Saenz, Luis A. Curiel-Ramirez, Javier Izquierdo-Reyes, Ricardo A. Ramirez-Mendoza

**Affiliations:** 1 School of Engineering and Science, Tecnologico de Monterrey, Mexico City 14380, Mexico; a01022495@itesm.mx (A.G.-B.); a01271635@itesm.mx (L.A.A.-S.); or l.curiel_ramirez@wzl.rwth-aachen.de (L.A.C.-R.); or javieriz@mit.edu (J.I.-R.); ricardo.ramirez@tec.mx (R.A.R.-M.); 2 Laboratory for Machine Tools and Production Engineering (WZL), RWTH Aachen University, 52074 Aachen, Germany; 3 Microsystems Technology Laboratories, Massachusetts Institute of Technology, Cambridge, MA 02139, USA

**Keywords:** smart mobility, V2I, fog computing, smart cities, intelligent transport systems

## Abstract

Analyzing data related to the conditions of city streets and avenues could help to make better decisions about public spending on mobility. Generally, streets and avenues are fixed as soon as they have a citizen report or when a major incident occurs. However, it is uncommon for cities to have real-time reactive systems that detect the different problems they have to fix on the pavement. This work proposes a solution to detect anomalies in streets through state analysis using sensors within the vehicles that travel daily and connecting them to a fog-computing architecture on a V2I network. The system detects and classifies the main road problems or abnormal conditions in streets and avenues using Machine Learning Algorithms (MLA), comparing roughness against a flat reference. An instrumented vehicle obtained the reference through accelerometry sensors and then sent the data through a mid-range communication system. With these data, the system compared an Artificial Neural Network (supervised MLA) and a K-Nearest Neighbor (Supervised MLA) to select the best option to handle the acquired data. This system makes it desirable to visualize the streets’ quality and map the areas with the most significant anomalies.

## 1. Introduction

Not all cities have the same problems, the same technological capacity or the same economic resources. That is why there are no concrete methods to solve the mobility of every urban environment, or there vehicle infrastructure problems. It is imperative to solve the city’s problems in the same context in which they occur and adapt the solution to the city’s resources and possibilities.

Mexico’s capital, Mexico City, as of 2020, is the home of 9,209,944 people [1]. With a city this enormous and territorially extensive, problems regarding mobility arise daily. Public transport inefficiencies, heavy traffic, and a deterioration of the urban landscape are just a few of the issues that millions of people must face every time they want to move through the city. These problems grow exponentially due to the poor city planning, and its accelerated growth rate, which limit emerging solutions. In the context of the available technology, the possibilities of implementation, and significant areas for improvement within the city, the current research aims to answer the long list of city problems under the specific scope of intelligent urban mobility. Furthermore, the proposed solution is aligned with affordability, effectiveness, attractiveness, and sustainability principles.

As our knowledge about mobility in the city increases, better proposals can solve the main issues. By measuring and intelligently gathering information regarding mobility and the condition of the vehicular infrastructures, we could provide a way to find areas of opportunity and implement efficient and effective solutions that could solve problems related to modern urban mobility in Mexico City. This work focuses on developing a platform that the government and road users can integrate into a sizeable vehicular network.

As we know it today, the Internet of Vehicles (IoV) focuses on the intelligent integration of humans, vehicles, things, and environments. It is a more extensive network that provides services for large cities or even a whole country. The IoV is an open, integrated network system with high manageability, controllability, operationalization, and credibility. It is composed of multiple users, vehicles, infrastructure, and networks [2,3].

Multiple applications arise from this technology. For instance, safety applications have been proposed in the past, such as notifying vehicles about dangerous situations within the roads ahead or identifying possible parking locations before the car arrives at the destination. The expectation is that vehicles can provide data about vehicle sensors, the environment, the driver, and passengers. A Road-Side Unit (RSU), or generally speaking, Road-Side Equipment (RSE), can exchange context-aware information with an On-Board Unit (OBU) or On-Board Equipment (OBE) installed inside any of the vehicles in the network. Context-aware information exchange between RSU/RSE and cars can help generate real-time information, e.g., traffic information [4].

Fog Computing is a highly virtualized platform that provides compute, storage, and networking services between end devices and traditional Cloud Computing Data Centers, typically but not exclusively located at the edge of the network. The defining characteristics of the Fog are: (a) low latency and location awareness, (b) widespread geographical distribution, (c) mobility, (d) a vast number of nodes, (e) the predominant role of wireless access, (f) strong presence of streaming and real-time applications, (g) heterogeneity [5].

There are many reasons why a fog computing architecture can benefit IoV communications, especially in V2I networks and infrastructures: The RSU can be physically damaged by some malicious activity or other harsh environments. With appliances exposed to weather, traffic, and all kinds of conditions, the cluster nodes will eventually go down.

The network connectivity during V2I and I2I communications may be temporarily cut off, affecting immediate data communication. The vehicular network should be sufficiently scalable to adapt to the increasing number of vehicles. As more and more people adopt vehicles with OBU capacities, or intelligent vehicles go out to market, the amount of nodes within clusters, and clusters themselves, must scale accordingly.

**Figure 1:**
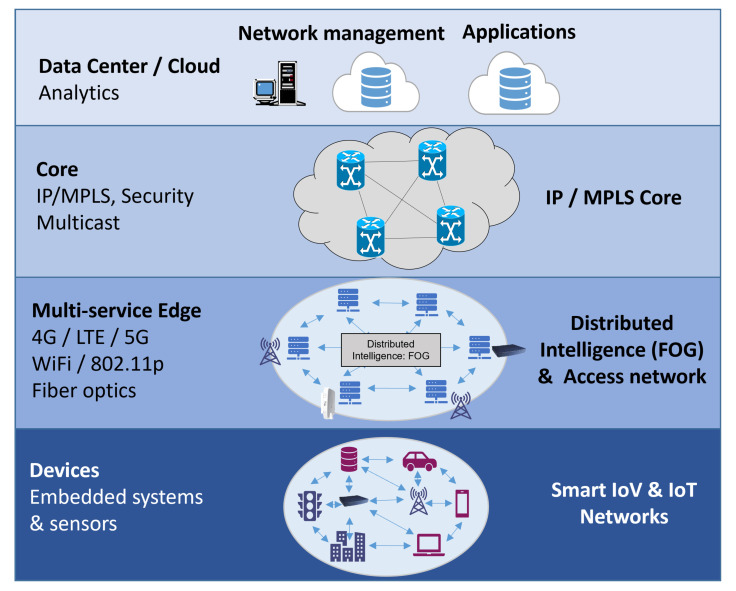
Fog Computing conceptualized architecture.

Based on this type of computing network, the architecture layer in the Figure 1 describes its need to separate data, communication, and fog cloud.

The evolution from local Vehicular Ad-Hoc Networks (VANETs), cloud-computing VANETS (VCC), and IoV to Vehicular Edge Computing (VEC), to, finally, Vehicular Fog Computing (VFC) has come a long way in the past two decades. A comparison of these architectures can be seen in Table 1.

With the enormous scalability of vehicular-fog-computing architectures, it is necessary to consider how to scale, create clusters, and take advantage of all the computing processing abilities acquired through these types of networks. While Edge Computing needs one suitable computing device at the border, Fog computing can be built with several low-cost computing devices as cluster nodes, making it more scalable. However, to surpass edge computing in terms of benefits, it must be built with the proper amount of nodes and clusters. The following section describes some state-of-the-art technologies that demonstrate the real benefits of such networks.

This work presents the context in which Mexico City currently operates regarding urbanism and mobility-related problems, technology, and communications. Moreover, an intelligent urban mobility approach is proposed to solve mobility problems using a V2I-fog computing architecture with data visualization tools on the cloud. At the same time, the solution aligns with the principles of affordability, effectiveness, attractiveness, and sustainability.

The technical goal of this work is to make possible the implementation of a solution focused on the identification and classification of road conditions and anomalies, such as potholes. With this architecture, it is possible to obtain a solution that integrates different sensors in vehicles, processes information at the edge of the network and detects problems in the streets through fog computing. Furthermore, data visualization through the cloud can provide valuable information to the city’s decision-makers.

## 2. Background

### 2.1. Smart Mobility and V2I Applications

When talking about smart mobility, the literature generally refers specifically to smart urban mobility. This distinction is essential because technology is completely city-oriented since most V2X networks rely heavily on QoS replication and are designed for high data transfer/processing volumes.

Even though multiple definitions frequently arise, this dissertation will take the one proposed by [6] as a reference, defining smart (urban) mobility as connectivity in towns and cities that are affordable, effective, attractive, and sustainable. These features are of particular value in the context of the following chapters; if for some reason a user cannot afford to connect to the infrastructure, the proposed infrastructure will not be smart enough.

Connectivity is only a means to an end. This end should be affordable, effective, attractive, and sustainable. However, this end should by no means strictly be on the high-end technological side, especially if it does not comply with any of the previous definitions of smart mobility. Several authors have condensed all the properties that a smart city should have to compare the smart mobility progress in a city. The Smart Mobility Indicator (SMI) proposed by [7] is an indicator based on technical infrastructure, information infrastructure, mobility methods, and vehicles used for this purpose and legislation. The technical infrastructure must be able to communicate with different kinds of transport. The quality of those communications and the implemented applications considerably impact the “smartness” of the city.

Main V2I applications found in the literature revolve around: traffic solutions, secure communications for data privacy, and data processing. Traffic signal algorithms to control flow traffic have been studied [8], where simulations have shown a decrease in waiting time to almost 80% and reductions in travel time of 15%, compared to non-connected vehicle scenarios.

B. LV et al. developed an LiDAR-Enhanced Connected Infrastructure to provide high-resolution traffic information to both smart vehicles, autonomous or not, and a central computer, providing up to 8 MB of data on real-time traffic in no more than 200 ms [9]. Communication security varies from safeguarding communications in the physical layer through mathematical models and algorithms with encoded transmissions [10], using blockchain technology as a way to secure information through V2I communications, with blockchain handling vehicle’s authorization [11,12].

Processing data with this type of architecture takes advantage of artificial intelligence. For example, autonomous vehicles platooning for time reduction using directional positioning algorithms in a platooning decision-making process with multiple communications technologies and sensors have been tested. Long Short-Term Memory (LSTM) models to reduce interference and enhance data streaming and performance can also be carried out in this type of network [13].

Multiple applications can depend on this type of technology, from secure safety data sharing [11] to enhance passenger user experience using deep reinforcement learning [14]. However, with the growing quantity of data generated at the edge, the speed of data transportation is becoming the bottleneck for the Edge-Cloud-based computing paradigm [15]. Since only one Gateway and processing node generally exist in edge computing networks, most of the computing goes to the cloud for further processing. VEC is an improvement from vehicular cloud computing networks. However, Fog Computing Networks (FCN) can be a better solution.

Some of the applications of short-range technologies vary from localization to warning signaling. One example of application in short-range ultra-wideband (UWB) technologies provides estimated ranges to track the vehicle’s position in an outdoor environment. Martin et al. built an accurate and reliable positioning solution based on the combination of UWB varying estimates and inertial and odometry data of the vehicle [16]. As it has a low cost and a long battery life, ZigBee technology Vehicle Identification is possible. One proposed application is the classification of vehicles and communication through the 802.15.4 protocol. In addition, Zigbee technologies can be useful in V2V communications under certain conditions for Collision warnings [15,17]. Other applications could include the communication of sensors in Wireless Personal Area Networks (WPAN) and low-energy, BLE-based fingerprint localization [18].

The applications of mid-range technologies are generally in the V2I-V2X spectrum, where data safety, management, and non-maximum security data processing are better suited.

Vehicle-to-vehicle communication in highways was tested in simulation with DSRC, but with high latency and packet error rates, proving the difficulties these technologies have with this communication type [19]. Other tested applications are vehicle communications in platooning scenarios [20] and maintaining lane distances with vehicle communications [21]. However, the results were never as promising, with higher latencies and packet error rates.

Pedestrian recognition, detection, and informing can also be considered within this range of applications [22], integrating human–vehicle classification through Support Vector Machines (SVM) and radar systems to the infrastructure while communicating this information to cars, bicycles, or even other pedestrians.

One sustainability application in the literature revolves around eco-driving with fuel optimization with traffic status and v2i communications [23]. The IEEE 802.11 family can disseminate the information and provide Internet access; however, if the goal of the network is to enhance complete autonomous driving, the integration of other technologies into the system is required [24]. Studies have been made about the support of applications over Wi-Fi in smart cities, testing access to the internet, or voice over IP (VoIP) applications, which can upgrade the user experience of passengers [25].

The applications of long-range technologies can achieve complete secure and low latency V2V and general V2X applications. Long-range technologies can make V2X service negotiation and location-aware scheduling possible to provide the network with insights into the vehicular equipment’s mobility pattern and application requirements, enabling the network to optimize the availability and scalability of automotive applications [26].

Another important benefit provided by 5G and C-V2X communications is a better Quality of Service (QoS). 5G offers a unique provision of computational resources and storage at the network edge, enabling the network to host applications closer to vehicles, reducing latency greatly. Multi-Access Edge Computing (MEC) efficiently utilizes the network core and backhaul, reducing the latency requirement of autonomous cars [27]. Since vehicular users are highly mobile, The system can support frequent handovers via resource management at the edge of the access network [26].

Most of these communications technologies operate within the IP protocol. Jeong et al. conducted a survey focused on different IP-based vehicular networks [28], discussing and comparing several protocols, architectures, and mobility handling techniques. An IP approach to vehicular communications is essential in a a cloud or fog computing environment to interconnect vehicles with the rest of the internet, combining all software applications.

### 2.2. Anomaly Detection within Smart Vehicles

The combination of communication technologies, fog computing, and different types of vehicular communications in a smart urban mobility environment provides the possibility of integrating with multiple kinds of Artificial Intelligence. Analyzing and classifying road conditions using Artificial Neural Networks (ANN) is not new, but these algorithms’ accuracy has improved.

One impressive feature that AI enables is driver’s behavior detection, including the detection of driving under the influence of alcohol [29] and detecting the driver’s emotional state through sentiment analysis [30], potentially saving drivers from accidents caused by their temporal inability to drive.

Besides the driver or passenger inside vehicles in V2X networks, artificial intelligence can detect other anomalies, i.e., pothole detection can be achieved using Convolutional Neural Networks (CNN) such as the AlexNet [31] or the YOLO CNN algorithm [32]. Furthermore, a combination of Long Short-Term Memory (LSTM) Neural Networks and CNN’s can detect data anomalies caused by faults or errors in the vehicle’s sensors, or even cyberattacks [33].

There are many approaches to detect anomalies in the streets via image recognition, and there are multiple approaches to sentiment analysis and behavioral classification and prediction. However, there was no literature on pothole detection through the sensors of an intelligent vehicle, or, vice-versa, sentiment analysis through image processing and CNN. While the latter could result in invasions of privacy, the former could potentially be a useful application for pothole mapping throughout the city.

Several investigations have been developed to identify and classify road conditions and their anomalies in technological systems. The research approach mainly differs in three points: acquisition of data, series of road anomalies and disorders due to detection or classification, and the algorithms applied to accomplish such tasks. Table 2 surveys and defines the main principles that guided the solutions in the last decade.

The proposed solution is that a V2I-Fog computing architecture that integrates different sensors in vehicles, and through fog computing and machine learning, detects problems in the state of the streets, will boost smart urban mobility solutions in terms of affordability, effectiveness, attractiveness, and sustainability. This solution needs to be broken down into several parts: Communications, RSU-OBU system, Machine Learning, and data visualization.

Several access points were distributed and connected through vendor-specific protocols to ensure communication between an OBU and an RSU. The OBU has three sensors and an external antenna device to acquire data from its surroundings and the data’s position. Then, through socket communications, it can send information to the RSU device. Thus, the RSU device handled both the server-side of the communication channel and the data preprocessing.

The third part corresponds to classification through Machine Learning. Machine learning algorithms have been proven to be the best alternative to usefully process information. Therefore, it is necessary to develop useful models that correctly classify the data obtained via the OBU. The last part corresponds to the visualization of the processed data, showing useful information regarding the conditions of the city’s streets and avenues. Once both MLAs have been tested and implemented, the algorithms will quantatively compare two streets to determine which road is the most troubled in terms of anomalies.

## 3. Experimentation

### 3.1. Communications & RSU

**Figure 2:**
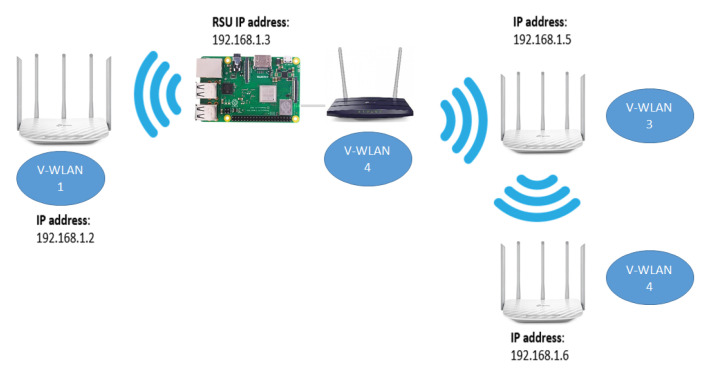
Full Network topology with Raspberry Pi as RSU and TP-Links as APs.

To test medium-range communications, several APs were wirelessly interconnected to form multiple LANs via WiFi. An RSU was connected via ethernet to the AP infrastructure to receive information from any potential OBU connected to the network. Each Access Point, OBU, and RSU was assigned a static IP Address, and the communication between the RSU and OBU was established using web Sockets with TCP protocol. Figure 2 shows a simple diagram of the network topology.

The RSU was built using a raspberry Pi, with the server’s software built-in with Python. Before the data were processed, they were saved in CSV format, with the latitude and longitude of each packet and a timestamp for future reference.

### 3.2. OBU

The OBU device was integrated with a Raspberry Pi 3B + as core, an MPU6050 accelerometer and gyroscope, a neo 6M GPS sensor, a camera module, and an external 6 dBm antenna for greater distance in communications. In addition, we completely programmed the system with Python 3.6, establishing a static IP socket communication to send the information from the OBU to any RSU device whenever there was a connection.

We carried out concurrent programming to ensure a better data-collection rate, and a buffer was implemented to obtain a significant amount of data while handovers or a wireless disconnection occurred.

The camera was only used for visual aid purposes, sending images every 200 datapoints to obtain a street-level photograph of the nearby location of an anomaly.

### 3.3. General Architecture

**Figure 3:**
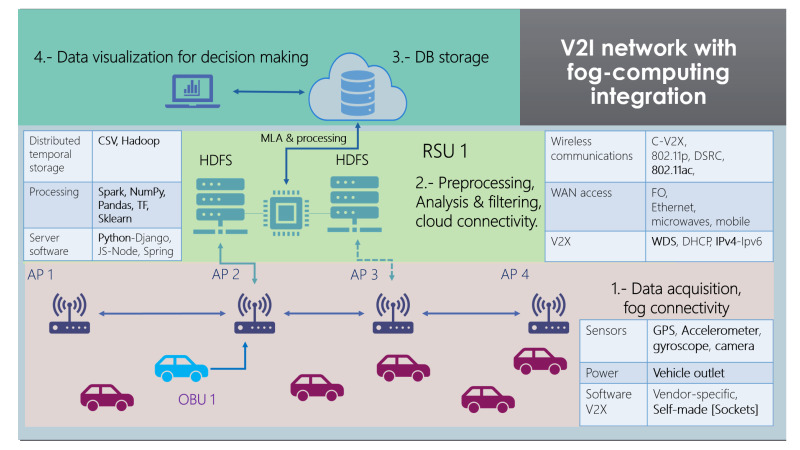
Fog Computing—V2I network proposed solution for Anomaly Detection.

With every component of the solution described above, a general graphic summary can be seen in Figure 3. The whole solution is divided into four sections, and each technology that was used for this fog computing architecture is mentioned in brief.

This architecture should be replicated throughout the city, with multiple nodes for each RSU and more OBUs to collect enough data mapping every street. This solution should be considered a cell, excluding all the processed and correctly classified data from the cloud database.

### 3.4. Experiment Setup

**Figure 4:**
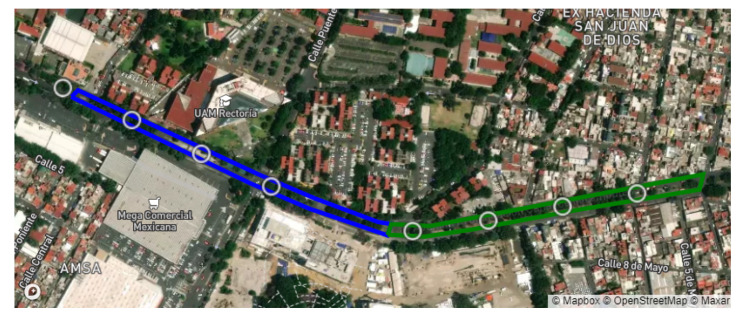
APs marks on the first route of the experiment.

The team selected two routes within the same avenue, called “Prolongación Canal de Miramontes”, in Mexico City. The total distance for the first route consisted of more than 1.1 km, while the second route was about 1 km long. The satellite view of both routes can be seen in Figure 4.

The experiment took place on a Friday afternoon, from 12 p.m. to 3 p.m., with light traffic on a sunny day, without any communication interference caused by the weather. This avenue had not been paved or maintained in more than ten years, and was full of anomalies. These included multiple potholes, unpainted speedbumps, defective traffic lights, and multiple U-turns and crosswalks. The speed limit for any vehicle is 40 kmph.

### 3.5. Data Acquisition for Reference

The experiment took place in different stages. First, for every route, the Access Points and RSU were placed according to Figure 4, respectively, and multiple laps were run for each route. Table 3 shows the summary of the data obtained by each experiment and the percentage of the route covered by the APs reached. This data were stored in the RSU for further processing.

The packet error rate of the proposed V2I network resulted in results of an average 2.47% PER, with a maximum of 8% in the worst configuration possible and unexpected anomalies in the communications, including interference, partial or complete obstruction in the line of sight, or unexpected weather conditions.

Additional data were acquired since the ANN model needs labeled data for training. This also obtained data for comparison. With new data on a less troubled street, the algorithms’ manual labeling of the anomalies and cleaner training data can be provided to the algorithm for a better performance.

**Figure 5:**
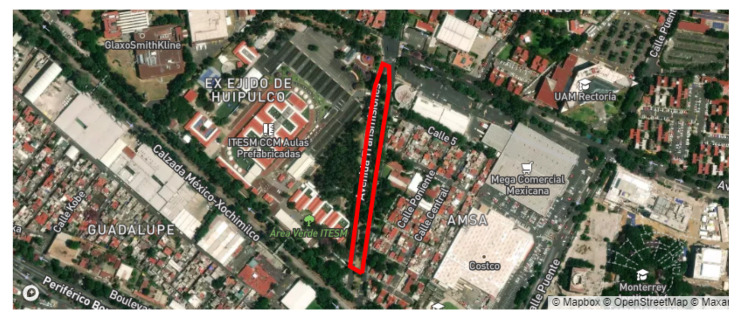
Satellite view of the reference street.

The street used as a reference had very few anomalies, was recently paved, and was mostly flat in all its extensions. “Transmisiones” street is located in front of a section of the Tecnologico de Monterrey—Mexico City campus. Therefore, it was a suitable candidate because it contained the aforementioned characteristics, and its proximity to the campus is a great advantage. In this whole street, we found three uneven drains, several speed bumps, and one steep curve; therefore, these were the labeled anomalies and were used as a reference in the training and classification of the visualization system. The Figure 5 shows a satellite view of the street.

“Transmisiones” street was traveled multiple times in order to read all the elements and anomalies in the street; eight unique physical anomalies were identified in the 0.7 km route, as seen in Figure 5. The pavement anomalies were detected at three different approximate average speeds: 20, 25, and 30 km per hour. Two access points were installed along the route, since we controlled the traveled and always measured within the coverage area; data acquisition was guaranteed along the route.

All samples were subsequently labeled by type of anomaly. For each of the 24 detected elements, a review of the event window was carried out. The definition of the window size was based on a graphical analysis of the time series plot of the measurements. The proposed windows consider a mean duration of each type of anomaly; a specific number of acceleration input samples was defined:

- Pothole: 60 samples
- Speed Bump: 95 samples
- Curve: 54 samples
- Plain: 84 samples

This was an effective method to label each one of the samples, by defining a “window size” for each one of the anomaly type. Given the number of detected anomalies and their corresponding window size, the resulting dataset contained 12,000 data points. Table 4 summarizes the classification of the acquired data.

We used these different datasets for model training. Nevertheless, before that, data processing was needed for performance reasons.

### 3.6. Machine Learning Classification Algorithms

Two models were proposed for the classification system, based on the type of data provided by the accelerometer: the K-nearest neighbor algorithm and an Artificial Neural Network. Even though both algorithms classify data, they are opposite approaches. The KNN algorithm is more practical and easier to train than the ANN, and ANN is a more sophisticated and likely better approach. However, considering the amount of experimental data obtained, the KNN could be better suited.

#### 3.6.1. KNN Algorithm and Architecture

Nearest Neighbor algorithms are among the simplest of all machine learning algorithms. The idea is to memorize the training set and then to predict the label of any new instance on the basis of the labels of its closest neighbors in the training set. The rationale behind such a method is based on the assumption that the features that are used to describe the domain points are relevant to their labels in a way that makes nearby points likely to have the same label [41].

The KNN algorithm is a classifier that considered N different kinds of neighbors to the sample, with a uniform weight consideration. This means that all points in each neighborhood are weighted equally. For this work, we built a KNN algorithm using Python’s library Sci-Kit Learn, considering four neighbors to our data input and receiving four output classes.

#### 3.6.2. ANN Algorithm and Architecture

An artificial neural network (ANN) is a model of computation inspired by the structure of neural networks in the brain. A neural network can be described as a directed graph whose nodes correspond to neurons and edges correspond to links between them. Each neuron receives as input a weighted sum of the outputs of the neurons connected to its incoming edges [41].

**Figure 6:**
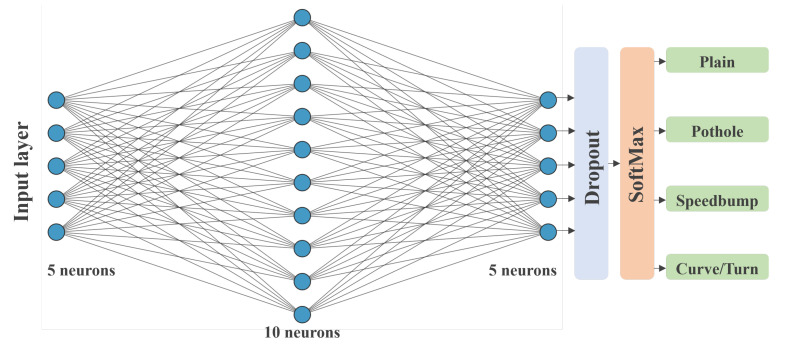
ANN network architecture.

The ANN was built using the TensorFlow library for Python. It is a sequential model with four hidden layers, and three dense hidden layers with five, ten, and five neurons, respectively. A dropout layer is used to reduce overfitting and improve the generalization of the network, and a Softmax layer is used to generate the four outputs. All fully connected layers used the Rectified Linear Unit (ReLU) activation function. The Figure 6 shows the full architecture of the neural network.

The team found that the neural network was rapidly overfitting the data after several training and testing routines. Therefore, we reduced the original number of neurons and added a Dropout Layer with a 0.15 rate in response to that behavior. This process ensured that the model behaved more accurately when presented with unknown data.

Data were divided into batches with sizes of 50 to train the network, and 15 epochs were set for backpropagation. While the batch size was arbitrary and iterated for performance reasons, a low number of epochs was set, given that the signals were identifiable, the training dataset was small, and overfitting was to be avoided. For the same reason as the small dataset, the validation data were not provided to the model. Two optimizers were tested to improve performance. The gradient descent with momentum, the Adam optimizer, and the differences were abysmal. The Gradient descent with momentum was unable to create an accurate model and, thus, was discarded.

ADAM is an algorithm for a first-order, gradient-based optimization of stochastic objective functions based on adaptive estimates of lower-order moments. The method is appropriate for non-stationary objectives and problems with very noisy and/or sparse gradients. Previous empirical results demonstrate that Adam works well in practice and compares favorably to other stochastic optimization methods. Given the nature of the data, a Categorical Cross-Entropy loss was used as the parameter that needs to be optimized.

### 3.7. Data Preprocessing Methodology

**Figure 7:**
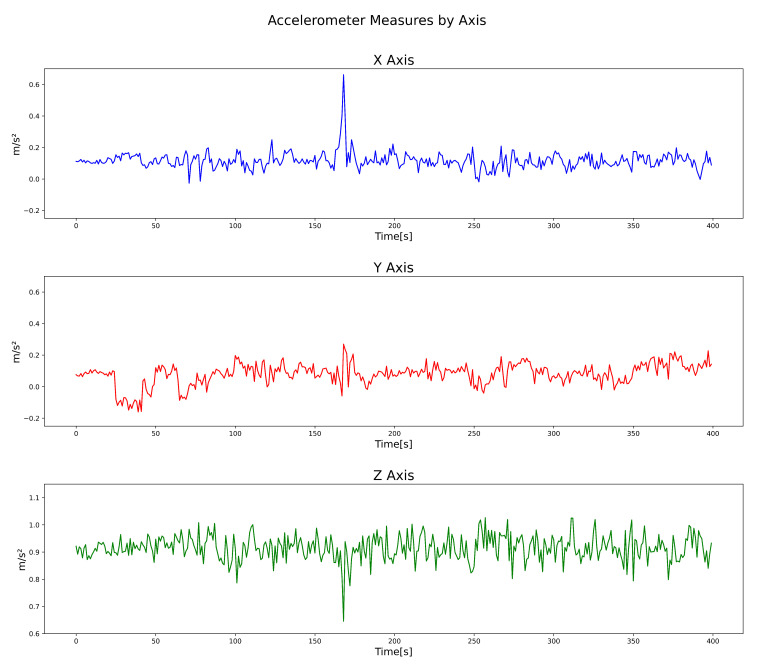
Street with pothole at 20 kmph on average.

Processing was carried out for every 50 datapoints that were obtained. For example, the Figure 7 shows an example of one signal containing one pothole, detected while driving at an average speed of 20 kmph.

**Figure 8:**
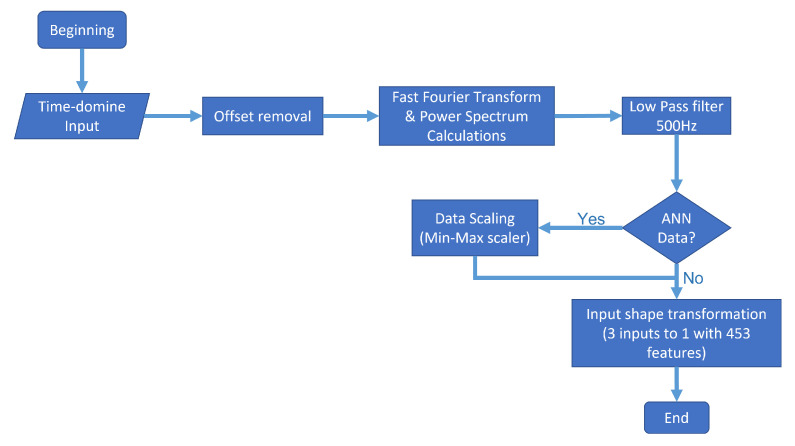
Data processing flow diagram.

In the example shown in Figure 7, from datapoint numbers 100 to 150, we conducted a process involving offset removal, Fast Fourier Transform, Power Spectrum calculation, low pass filtering, and, depending on the model, an extra step for scaling, and matrix transformation from 151 × 3 to 453 × 1. The Figure 8 shows a flow diagram of the entire process.

1. *Offset removal*. Given the nature of the sensors and the practically constant gravitational force in the accelerometer’s z-axis, we decided to eliminate the signal’s offset by subtracting the mean in every axis. Thus, even though a DC component in a frequency spectrum analysis is irrelevant, as it represents a peak around 0 Hz, it represents noise in the input of both models. Therefore, we decided to remove it.
2. *Time-domain to frequency-domain transformation*. Based on the power spectrum algorithm via an FFT transform, the signal of every axis was transformed into a power spectrum, and every frequency superior to the 500 Hz threshold was ignored, since the sample rate of the sensor was 1 kHz.
3. *Data were scaling*. In the case of the artificial neural network, additional scaling was performed to improve the training process using a min–max scaler from 0 to 1. This process is because scaled data tend to improve training, given the high number of matrix multiplications by the nature of the artificial neural network. This characteristic is the main difference between the input of both models. On the other hand, the KNN algorithm classifies information based on different types of distances: Euclidean distance, cosine squared distance, etc. Moreover, the scaling of data does not represent any difference.
4. *Input shape transformation*. For every 50 × 3 datapoints provided by the OBU, the signal was repeated five times before any further processing to more easily show the power peaks in the signal. Finally, we removed the offset, the power spectrum was obtained, and a single sample with 453 total features was set as the neural network’s input and the KNN ML algorithm.

**Figure 9:**
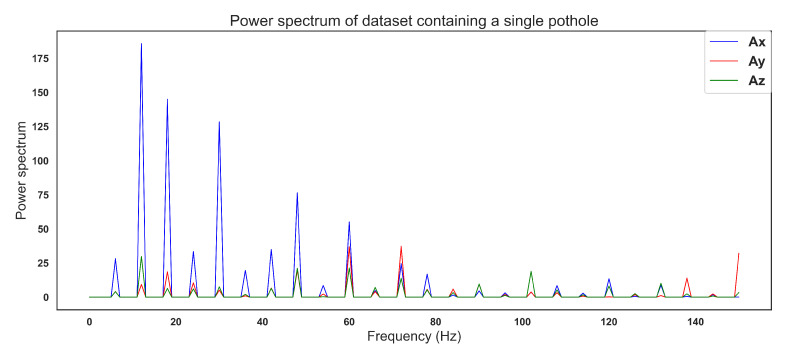
Power spectrum of a pothole.

After this process, the signal Figure 7 finally transformed into a Figure 9, where the energy contained in different peaks throughout the signal can be seen.

All the data obtained during the experimentation phases underwent this treatment before moving on to the training and testing phase of the machine learning algorithms.

## 4. Results

Both algorithms were trained, with highly acceptable results. Both algorithms had over 90% accuracy during training, with the KNN having 95.55% accuracy and the ANN scoring 96.79% over the training data.

**Figure 10:**
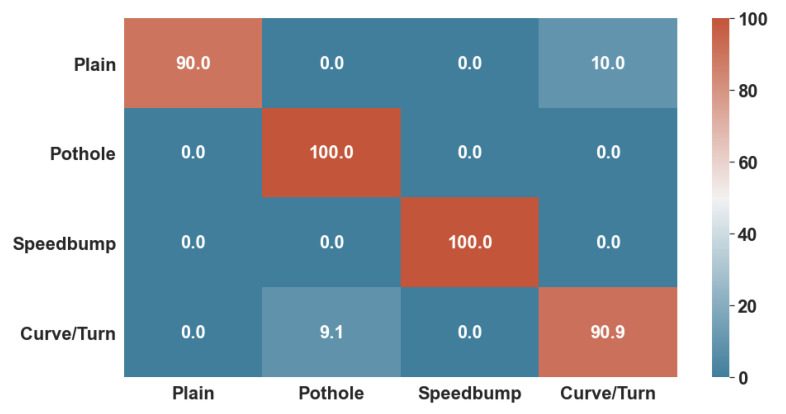
KNN Confusion Matrix over Test Data.

**Figure 11:**
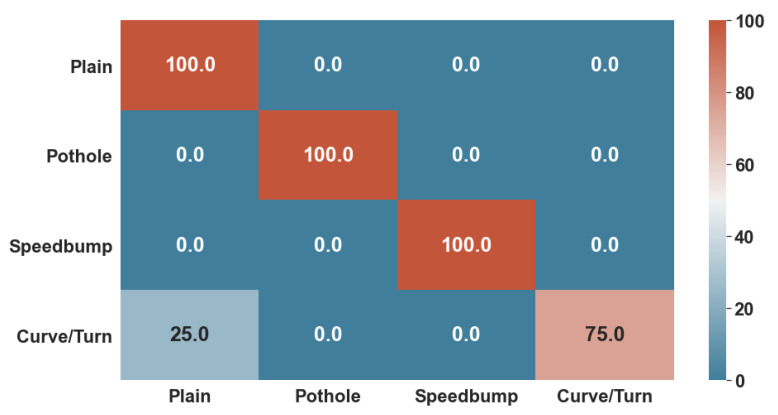
ANN Confusion Matrix over Test Data.

Figure 10 and Figure 11 show the confusion matrices generated with the test data. While the KNN algorithm did have minor errors in classifying non-anomalous data and curves, the ANN had trouble classifying only curves. Thus, the number 0 represents healthy data; number 1 represents possible potholes, number 2 speed bumps, and number 3 harsh curves.

Lastly, both algorithms were given 85% of the whole dataset to train and 15% to test. These tests consisted of accuracy for training, and F1-Score for test data, as well as a confusion Matrix, to determine the specific classifications in which they did not perform well enough.

The other measurement was the F1 score, which measures complete accuracy using both precision and recall. The score reaches its best value at one and its worst at 0. The precision refers to the number of true positives per true positive and false positive, while the recall measures the number of true positives per true positives and false negatives.

$$Precision=\frac{TruePositives}{TruePositives+FalsePositives}$$

$$Recall=\frac{TruePositives}{TruePositives+FalseNegatives}$$

$$F1score=2\times \frac{Precision\times Recall}{Precision+Recall}$$

**Figure 12:**
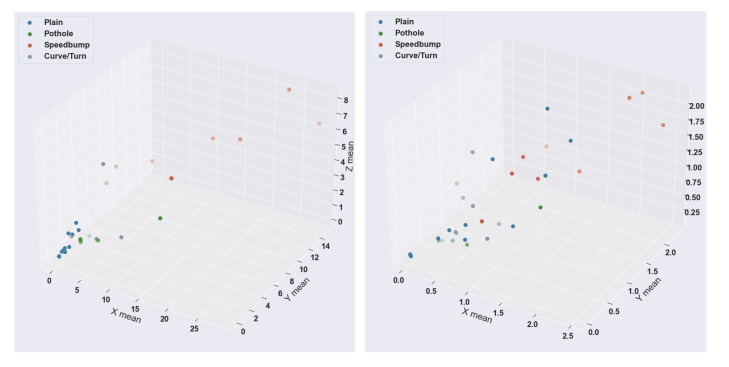
KNN 3D scatter plot (**left**) vs ANN 3D scatter plot (**right**).

The results on the test data were coherent with the results of the training. For example, the KNN algorithm scored 97.80%, while the ANN scored 93.33%. To visualize the presented data, we made 3D scatter plots with the labeled test data. Figure 12 shows both scatter plots, with the mean power of the frequencies for the three axes of the sensor data. The label is the same as the confusion matrices, with four different colors. It can be seen in the 2D plot of a particular case in Figure 9. Both results have a complete similarity, and the types of anomalies that differ the most within these visualizations are speed bumps and good road data. The dataset was randomized from the rule of 85% for training and 15% test; thus, each algorithm received different test datapoints. That is why the points are different in each scatter plot, but we can correctly observe the color classification of the output of each algorithm.

### 4.1. Street Anomaly Comparison

**Figure 13:**
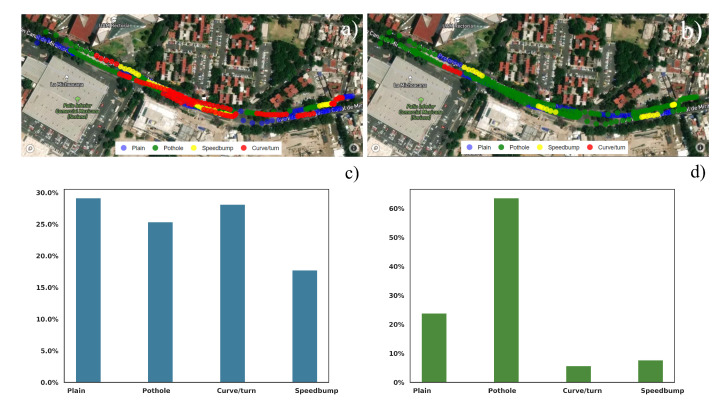
Geo spatial representation of the different anomalies by the KNN algorithm. (**a**) KNN (**b**) ANN. Barchart of the various anomalies classified by the ANN algorithm by percentage (**c**) KNN (**d**) ANN.

There were two different streets in which we collected data. On the first street, with fewer anomalies, the KNN algorithm classified the various abnormalities correctly compared with human observations. Figure 13a,b shows the visual representation of the anomalies, while Figure 13c,d shows a barplot of the percentage of each category.

On the other hand, the ANN algorithm somehow failed to correctly classify the different anomalies on the street. As a result, there are many misclassified potholes on the road, and the number of healthy and anomalous streets is not coherent. Figure 13b shows the map with the classified anomalies by the ANN, while Figure 13d shows the bar chart.

The second street has a lot more anomalies and troubles. Since there are many anomalies in a bigger proportion and magnitude, these data are more prone to error. However, it is important to compare the amount of healthy street distance versus data containing one or more anomalies.

### 4.2. Visualization

We carried out the same preprocessing for each model with two different datasets: one composed exclusively of random sections of the reference dataset and another with multiple laps from the experiment. The idea is to completely visualize how the streets would look and be classified with these models.

The KNN classifier worked in a very acceptable way, as Figure 13a. While the system correctly detected the reference avenue, all the data obtained from the experiment seem approximately correct. The red zones Figure 13a results are coherent with the observations, and we specifically chose that avenue because of the number of anomalies presented.

The ANN, on the other hand, incorrectly lebeled the street where the model was trained and seemed to mislabel some of the information of the experiment’s data. Figure 13b shows significant differences with the KNN classifier.

Geospatial data with color-based anomaly representation were programmed with the help of Python’s library Folium. These maps were made to compare the streets within Mexico City and were not implemented in a web application.

Geospatial visualization was carried out to compare and choose a better model for classifying anomalies on the city’s streets. The main reason why KNN was a better fit was that it did not require data scaling for its inputs. Thus, the KNN accurately handled a street with deep potholes, speed bumps without any paint or signaling, and a generally more chaotic avenue that generated bigger sensor signals.

Maps generated with the folium library can be exported. Since they run a technology called leaflet as the underlying software, they can be exported or even developed with front-end technologies, libraries, and frameworks such as React, Angular, Vue, or any technology that runs with the Node runtime environment.

The KNN classifier’s most common issue was mislabeling other classes, or neighbors, into curves. However, with only one physical example, data with a more varied power spectrum were more likely to fall into that category. The ANN classifier failed to accurately detect much of the data without distinction. One of the main problems of the classifier could be the sensitivity to scaling.

The ANN showed an unconvincing probability for each of the classes in most cases, and scaling could potentially be the main issue in the classification. Since the anomalies were more frequent, greater, and different from the training dataset, the amplitude of the bigger anomalies was likely difficult to translate into correctly scaled signals for the ANN to recognize.

Even though both algorithms were trained in a completely similar form, and the test of both algorithms resulted in great performance results, the data obtained from the street reference were not completely identical to the experiment’s data, as shown in the classification result. A deeper analysis of this situation is presented in Section 5, but there are several reasons why this happened, including labeling, human error, and lack of training data.

## 5. Discussion

Three factors heavily influenced the experiment, and can substantially improve the prediction in real scenarios. The first factor is the amount of data collected, the second is the quality of the road, and the third is the different road characteristics that exist in real scenarios.

For a low-complexity MLA, the data may be sufficient to obtain an acceptable prediction. However, for any DeepLearning solution, the data are frankly insufficient to take advantage of this type of algorithm. In addition, the number of anomalies is very limited due to the lack of a classification of anomalies and healthy streets’ data and the volume of data that are necessary to define clear differences between each of them from the spectral footprints.

During the data acquisition process, the vehicle did not experience unevenness with great height differences, nor did it pass through tunnels, bridges, traffic lights, or other typical situations for a city road. Thus, this limited its ability to differentiate common cases of healthy streets and anomalies such as bumps, potholes, or any other type. Therefore, it is necessary to include all kinds of daily situations that do not represent a problem for the road in the training.

In addition to these situations, it is possible to find deeper details within the same anomalies, such as potholes of different depths, speedbumps of various types, etc. Therefore, the anomalies can be subclassified to obtain a clearer representation of the data.

This system also has limitations related to the dynamics of the vehicles, since the oscillations generated by the vehicle at a movement greater than 35 km/h generated enough noise to make the anomalies undetectable. This very low maximum speed significantly limits the use cases of the system.

## 6. Conclusions and Future Work

This system proved to detect anomalies and create geospatial data visualization to represent the condition of the streets on two tested avenues. While the proposed research hypothesis proved true, this project has vast areas of opportunity, potential improvements, and further research to scale a system to a whole-city level. One of the new aspects of this project is the cloud solution. The design and implementation of cloud services to host the website are needed for the visualization tools and the creation and maintenance of a database, relational or not, as well as the complementary systems necessary for the operation of the proposed data recovery and analysis platform.

More data should be acquired and more datasets generated, with more labels and more precise labels for anomaly detection, including more anomalies, such as culverts, transverse and longitudinal cracks, stoplights, speed bumps, and dents, etc. The acquisition could be made in the periphery of Tecnologico de Monterrey Mexico City Campus before being expanded to other neighborhoods or counties.

Experiments, training, and more tests are required for the MLA proposed in this dissertation, with more data, better labeling, and a fog-cloud solution. This project proposed a simple geospatial visualization tool for anomaly detection. However, the most appropriate, efficient, and useful visualizations for observing relevant information on detected anomalies and areas of opportunity found in the data that were retrieved by the OBUs have yet to be determined.

Finally, this project could automatically express recommendations and conclusions regarding urban mobility to the corresponding authorities, instead of only showing visualization.

Other recommendations for further research include the analysis of the deployment of 5G in upcoming years, joining different network alternatives for different types of traffic, software-defined networks, and research into other virtualization tools for better V2I network scalability.

## Figures and Tables

**Table 1:** Comparison between VCC, VEC, and VFC architectures.

Features	VCC	VEC	VFC
Location	Remote location	User’s proximity	User’s proximity and remote location
Latency	High	Low	Low
Mobility support	Limited	Higher	Highest
Decision Making	Remote	Local	Remote & local
Communication	Constraints in Bandwidth	Real-Time	Real-time and asynchronous
Storage Capacity	Highly scalable	Limited	Highly scalable, both locally and remotely
Context Awareness	No	Yes	Yes
Device Heterogeneity	Limited	Highly supported	Highly supported
Computing Capability	High	Medium	High
Cost of Development	High	Low	Medium

**Table 2:** Main research approaches for the detection of road anomalies.

Authors	Data Acquisition Technique	Anomalies and Conditions	Data Analysis Technique	Results
Bhat et al., 2017 [34]	Gyroscope and accelerometer data, speed, and GPS location of the vehicle, using two iOS applications.	Classify “pothole” and “non-pothole”, as well as the good or bad road conditions.	Data are grouped into intervals to reduce noise. Use of SVM model to classify good-bad roads and predict potholes.	Road condition: 93.4% accuracy Pothole detection: 78% accuracy and 42% recall Models deployed on a cloud-based web server
Zheng et al., 2019 [35]	Legacy datasets, simulated data through Carsim^®^	Detect potholes, speed bumps, and metal bumps.	QF-COTE. Threshold detection and sliding window algorithm.	Method fitted to be used under an edge computing schema.
Pawar et al., 2020 [36]	Accelerometer and gyroscope data from smartphone mounted on the windshield	Pothole occurrences	Use of a Neural network based on ReLU activation function.	94% of accuracy and 81% of recall were reported. Suitable for real-time systems.
Wu et al., 2020 [37]	Accelerometer and GPS data obtained through a purpose-built mobile application.	Classify the general condition of the road: “normal road” and “pothole”	Training of various machine learning classification models. Wavelet, time and frecuency domain data used as input.	Precision and recall rate excede 95%
Wang et al., 2015 [38]	Mobile sensing, through accelerometer data normalization	Pothole detection	Implementation of an algorithm based on dynamic threshold detection, using three-axis accelerometer data.	100% accuracy, limited by the sample size used in the research.
Mednis et al., 2011 [39]	Preliminary data gathered from a modified LynxNet collar device.	Large and small potholes, clusters of potholes, gaps, drain pits.	Set of algorithms based on accelerometer data and threshold definition: Z-THRESH, Z-DIFF, STDEV(Z), G-ZERO	Algorithms deployed on limited hardware/software devices. True positive rates as high as 90%
El-Wakeel et al., 2018 [40]	Multiple IMUs, GPS receivers, smart devices, low-cost MEMS, mounted on a testbed.	Potholes, maintenance holes, transverse cracks, longitudinal cracks, railroad tracks, speedbumps, deceleration strips, paved roads, and road dents.	The obtained signals were de-noised using wavelets, and data sets are “time windowed”, The algorithm applied feature extraction techniques.	Multi-level SVM classifier, average TPR performance of 90%

**Table 3:** Experiment routes specifications.

Route	Laps	Sent Packets	Total Distance [m]	Route Coverage [%]
First route	5	2182	1120	92.6%
Second route	3	1414	997	50%

**Table 4:** Classification of acquired data.

Type	Unique Physical Anomaly	Total Physical Samples	Total Individual Sensor Samples
pothole	3	9	3000
speed bump	2	6	4800
curve	1	3	2700
plain	2	6	4200
Sum	8	24	12,000

## Data Availability

Not applicable.

## Abbreviations

The following abbreviations are used in this manuscript:

5G	Fifth Generation Technology Standard for Broadband networks
AI	Artificial Intelligence
ANN	Artificial Neural Network
AP	Access Point
CNN	Convolutional Neural Network
C-V2X	Cellular-V2X
DSRC	Dedicated Short Range Communications
FCN	Fog Computing Networks
FFT	Fast Fourier Transform
IEEE	Institute of Electrical and Electronics Engineers
IoV	Internet of Vehicles
IP	Internet Protocol
KNN	K-nearest neighbors
LSTM	Long Short-Term Memory
MEC	Multi-access Edge Computing
MLA	Machine Learning Algorithm
OBU	Onboard Unit
QoS	Quality of Service
RSU	Roadside Unit
SVM	Support Vector Machines
VANET	Vehicular Ad-Hoc Network
V2I	Vehicle to Infrastructure
V2V	Vehicle to Vehicle
V2X	Vehicle to Everything
VCC	Vehicular Cloud Computing
VEC	Vehicular Edge Computing
VFC	Vehicular Fog Computing
VoIP	Voice over IP
Wi-Fi	Wireless Fidelity
YOLO	You Only Look Once
